# Artificial intelligence applications in the management of musculoskeletal disorders of the shoulder: A systematic review

**DOI:** 10.1002/jeo2.70248

**Published:** 2025-04-28

**Authors:** Umile Giuseppe Longo, Martina Marino, Guido Nicodemi, Matteo Giuseppe Pisani, Jacob F. Oeding, Christophe Ley, Rocco Papalia, Kristian Samuelsson

**Affiliations:** ^1^ Fondazione Policlinico Universitario Campus Bio‐Medico Roma Italy; ^2^ Research Unit of Orthopaedic and Trauma Surgery, Department of Medicine and Surgery Università Campus Bio‐Medico di Roma Roma Italy; ^3^ Department of Orthopaedics, Institute of Clinical Sciences, Sahlgrenska Academy University of Gothenburg Gothenburg Sweden; ^4^ Department of Mathematics University of Luxembourg Esch‐sur‐Alzette Luxembourg; ^5^ Sahlgrenska Sports Medicine Center Gothenburg Sweden

**Keywords:** artificial Intelligence, machine learning, orthopaedic surgery, shoulder disorders

## Abstract

**Purpose:**

The aim of the present review is to evaluate and report on the available literature discussing artificial intelligence (AI) applications to the diagnosis of shoulder conditions, outcome prediction of shoulder interventions, and the possible application of such algorithms directly to surgical procedures.

**Methods:**

In February 2024, a search of PubMed, Cochrane and Scopus databases was performed. Studies had to evaluate AI model effectiveness for inclusion. Research on healthcare cost predictions, deterministic algorithms, patient satisfaction, protocol studies and upper‐extremity fractures not involving the shoulder were excluded. The Joanna Briggs Institute Critical Appraisal tool and the Risk of Bias in Non‐randomised Studies of Interventions tools were used to assess bias.

**Results:**

Thirty‐three studies were included in the analysis. Seven studies analysed the detection of rotator cuff tears (RCTs) in magnetic resonance imaging and found area under the curve (AUC) values ranged from 0.812 to 0.94 for the detection of RCTs. One study reported Area Under the Receiver Operating Characteristics values ranging from 0.79 to 0.97 for the prediction of clinical outcomes following reverse total shoulder arthroplasty. In terms of outcomes of rotator cuff repair, an AUC value ranging from 0.58 to 0.68 was reported for prediction of patient‐reported outcome measures, and an AUC range of 0.87–0.92 was found for prediction of retear rate. Five studies evaluated the identification of shoulder implant models following TSA from radiographs, with reported accuracy ranging from 89.90% to 97.20%.

**Conclusion:**

AI application enables forecasting of clinical outcomes, permits refined diagnostic evaluation and increases surgical accuracy. While promising, the translation of these technologies into routine clinical practice requires careful consideration.

**Level of Evidence:**

Level IV.

**Abbreviations**
AI: artificial intelligence
ASI: anterior shoulder instability
AUROC: area under the receiver operating characteristics
CDST: clinical decision support tool
CNN: convolutional neural network
ML: machine learning
PROM: patient‐reported outcome measure
RCR: rotator cuff repair
RCT: rotator cuff tear
RF: random forest
SITC: supraspinatus/infraspinatus tendon complex
SVM: support vector machine
TSA: total shoulder arthroplasty
(SLAP) lesion: superior labrum anterior to posterior

## INTRODUCTION

The shoulder is susceptible to a variety of musculoskeletal disorders, affecting physical function and quality of life, with biological factors contributing to its pathogenesis [30, 33]. Traditional diagnostic, prognostic and treatment methods have limitations in precision and personalization. Artificial intelligence (AI), especially through machine learning (ML) models, offers a data‐driven, algorithm‐enhanced approach for addressing these disorders in orthopaedics.

AI is transforming the field of orthopaedics through diagnostic, predictive, and surgical models. Diagnostic AI interprets data sets such as imaging, patient‐reported outcomes, and clinical assessments to enhance injury risk estimation and early detection of conditions like rotator cuff tears (RCTs) and superior labrum anterior posterior (SLAP) lesions [2, 15]. Predictive AI analyses clinical and demographic data to forecast post‐operative complications, length of stay, and patient‐reported outcomes, enabling personalized treatment adjustments to optimize recovery [7, 8, 9, 10]. In the surgical field, AI aids in implant identification from post‐operative imaging, enhances preoperative planning with 3D visualizations, and improves patient engagement through virtual tools, ultimately refining decision‐making and communication in procedures like total shoulder arthroplasty (TSA) [6, 21].

However, while AI's potential in orthopaedic surgery continues to grow, its specific effectiveness and limitations in shoulder‐related applications remain unclear [45, 46, 47, 48]. Indeed, a detailed synthesis of evidence on their effectiveness and limitations when applied to the shoulder joint is absent.

The aim of the present systematic review is to evaluate and report on literature discussing AI applications to the diagnosis of shoulder conditions, outcome prediction of shoulder interventions, and the possible application of such algorithms directly to surgical procedures.

## MATERIALS AND METHODS

### Eligibility criteria

A systematic review of the literature was carried out in February 2024. Full‐text articles written in English or Italian were included and encompassed both prospective and retrospective studies, as well as case‐series and case–control studies. The review focused on adult populations (>18 years of age). The studies selected for inclusion were those that investigated the capability of ML to forecast outcomes of orthopaedic procedures on the shoulder, predict diagnoses, or enhance surgical precision. Review articles, non‐clinical studies, editorials, letters to editor, protocol studies, and all articles that used deterministic algorithms or AI to predict the cost of healthcare, were excluded. Moreover, articles that measured satisfaction as an output variable and those investigating upper extremity fractures not involving the shoulder joint were not included. To be included, articles had to assess at least one of the following outcomes: area under the receiver operating characteristics (AUROC)/area under the curve (AUC), accuracy, F1 Score, precision, sensitivity, recall, specificity, Brier Score, C‐Statistic or patient‐reported outcome measures (PROMs). Studies focusing on applying AI algorithms to imaging were considered for inclusion.

### Information sources

**Figure 1:**
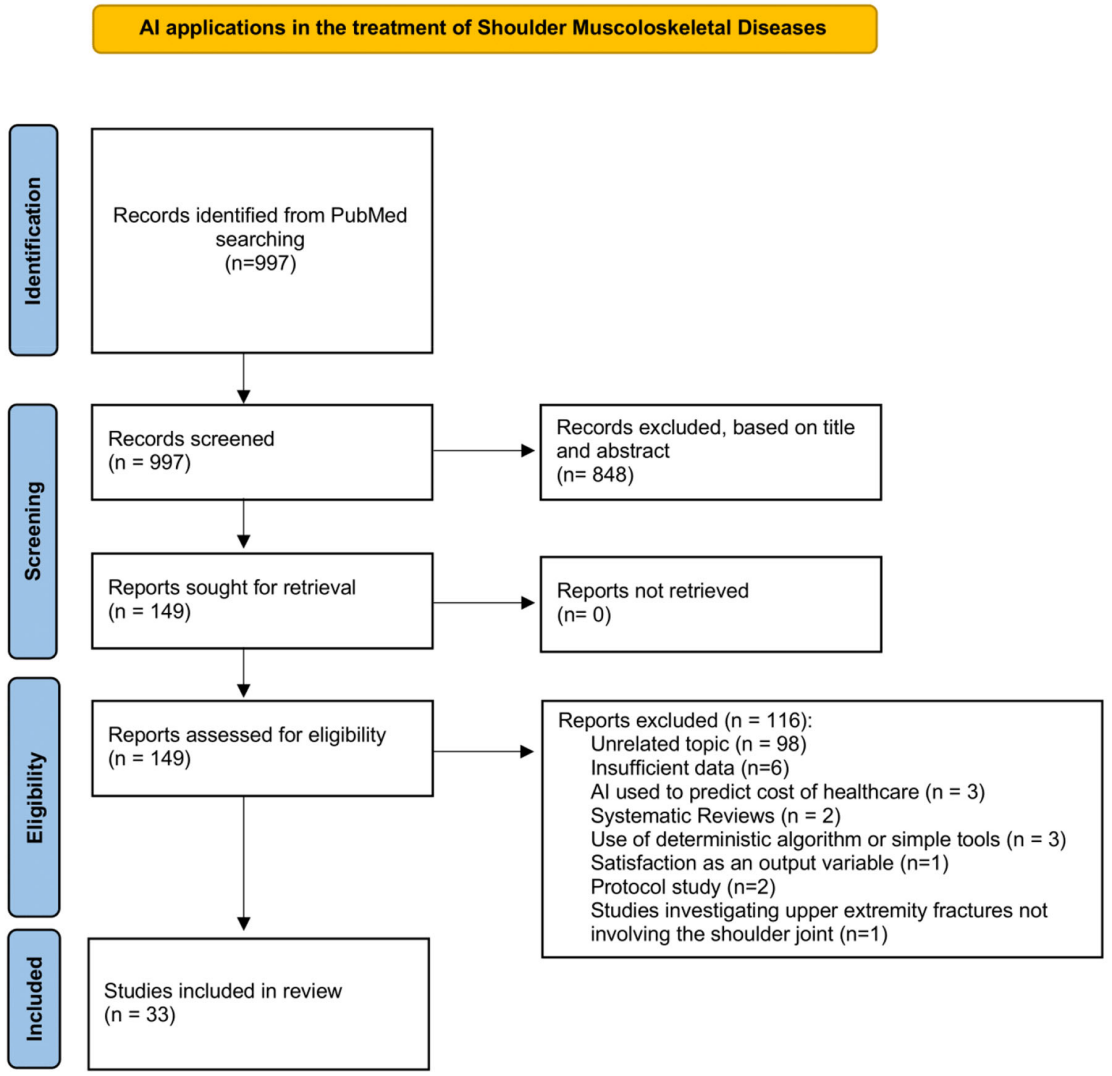
PRISMA flow chart depicting selection of included studies. PRISMA, Preferred Reporting Items for Systematic Reviews and Meta‐analysis.

A systematic literature search of the following bibliographic databases was completed: the US National Library of Medicine (PubMed/MEDLINE), SCOPUS, Cochrane Database of Systematic Reviews, EMBASE with no data limit. The search was performed following the Preferred Reporting Items for Systematic Reviews and Meta‐analysis (PRISMA) guidelines [40] (Figure 1).

### Search strategy

To carry out a literature search grounded in evidence‐based practice, the Population, Intervention and Outcome (PIO) framework was utilized. Patients affected by pathologies of the shoulder or undergoing related surgical intervention were considered (P) and applications of AI (I) to diagnosis, outcome prediction and surgical enhancement were evaluated. Outcomes of AI application were reported (O).

The search strategies used a combination of Medical Subject Heading (MeSH) terms and “title/abstract” search. For each database the exact search strategy can be found in Appendix A. No further search strategies were used.

### Selection process

Title and abstract screening were initially performed by two independent reviewers (GN and MGP), the same reviewers performed full‐text screening of the selected articles to verify if they met the eligibility criteria. Differences or disagreements at any stage were resolved with consultation of a third reviewer (MM); if doubts persisted the other contributing authors were consulted.

### Data collection

After the selection of eligible studies, data was extracted, including the name of the first author, year of publication, study design, level of evidence, aim, participant characteristics (sample size, mean age, gender distribution, type of disease and type of intervention), shoulder disease, type of intervention, ML model and outcomes of ML application. For studies with incomplete data or with data that was not directly detectable, an initial attempt was made to contact the corresponding author for feedback. In case of non‐response or inability to provide additional data, other reviews were checked to verify the presence of the data of interest published in other systematic reviews. The collected studies have been stratified into three distinct classifications based on their application domain within the realm of AI: predictive analytics (Table 1), diagnostic algorithms (Supporting Information: Table S1), and AI‐assisted surgical precision enhancer (Supporting Information: Table S2).

**Table 1:** Results of studies evaluating diagnostic artificial intelligence algorithms.

Author, Year	Study design	Level of evidence	Sample size	Mean age (SD)	Female patients %	Shoulder disease	Type of intervention	Machine learning model	AUC of ROC curve	Accuracy	F1 score	Precision	Sensitivity	Specificity
Alike, 2023	Retrospective Case‐series study	IV	974 patients	49.94 ± 14.2	52.17%	SITC injuries	/	Lasso	0.832	0.763	/	/	0.893	0.667
			Training cohort: (*n* = 828)					SVM	0.866	0.757			0.933	0.627
			Internal validation: (*n* = 89)					DT	0.826	0.756			0.759	0.753
								RF	0.897	0.829			0.783	0.863
								ResNet‐101	0.753	0.678			0.793	0.596
									0.788	0.718			0.827	0.637
								VGG‐19	0.753	0.678			0.793	0.596
								Inception‐V3	0.797	0.712			0.8	0.653
			External validaiton: (*n* = 57)					Ensemble DL model Ensemble CMD‐DL model	0.902	0.836			0.88	0.812
Guo, 2023	Prospective	II	770 patients	50.38 ± 17.78	46.88%	Supraspinatus Tear	Shoulder Arthroplasty	2D CNN	0.921	0.87 (STS)	0.82 (STS)	0.75 (STS)	0.91 (STS)	0.84 (STS)
	Case–control study		Retrospective Training Set: (*n* = 701)					3D CNN based on Xception	/	0.78 (ITS)	0.69 (ITS)	0.67 (ITS)	0.73 (STS)	0.81 (ITS)
			Prospective Validaiton Surgical Set: (*n* = 69)											
Kang, 2021	Retrospective	III	3746 patients	60.94 ± 11	56.72%	Subcapularis tendon teart	Arthroscopic surgery	Multimodal DL model	0.83	0.83	/	/	0.776	0.996
	Case–control study		X‐Ray: (*n* = 3119)											
			MRI: (*n* = 627)											
Kyu‐Chong Lee, 2023	Retrospective	III	794 patients	59 ± 11	52.89%	RCT	/	YOLOv8, FPN, PAN Combined Evaluating:						
	Case–control study		(694 RCT and 100 no RCT)					All	0.94	0.96	0.97	0.98	0.98	0.91
								Axial	0.71	0.58	0.68	1	0.51	1
								Sagittal	0.70	0.70	0.81	0.92	0.72	0.63
								Coronal	0.68	0.55	0.64	0.98	0.48	0.95
Su Hyun Lee, 2023	Retrospective	IV	303 patients	64.5 ± 8.2	51.81%	Full‐thickness RCT	/	U‐Net CNN	/	0.943	0.905	0.849	0.971	0.95
	Case‐series study													
Li, 2023	Retrospective	III	1684 patients	49.8 ± 14.4	53.30%	RCT	Arthroscopic surgery	Staking	0.9	0.81	/	/	/	/
								Bagging	0.92	0.83				
								AdaBoosting	0.75	0.81				
								GB	0.87	0.81				
	Case–control study		(417 RCT and 1267 other diseases)					RF	0.91	0.83				
								XGBoost	0.92	0.85				
Mu, 2021	Prospective	III	8 groups of patients	/	/	/	/	Alex Net	/	0.925	/	/	0.925	/
	Case‐series study		100 MRI Images					VGG16		0.905			0.905	
								Inception 3		0.911			0.911	
								ResNet		0.915			0.915	
Ni, 2023	Retrospective	III	Data set 1: 514 patients	61.5	43%	Class 0 = normal (*n* = 396)	Arthroscopic surgery	DL SLAP‐Net	0.98	0.96	/	/	0.94	1
			MRI Scanner: Discovery 750 W Silent			Class 1 = SLAP lesion (*n* = 240)			0.98	0.96			1	0.94
			Data set 2: 122 patients			Normal (*n* = 396)		DL SLAP‐Net	0.92	0.85			0.90	0.76
	Case–control study		MRI Scanner: Magnetom Trio and UMR 770			SLAP lesion (*n* = 240)			0.92	0.85			0.76	0.90
Oeding, Pareek, 2024	Retrospective	III	202 patients	59.1 ± 10.3	43.10%	Subcapularis tendon teart	ARCR	XGBoost	/	0.85	0.87	/	/	/
	Case–control study		Partial or full thickness TSS (*n* = 121)											

Abbreviations: ARCR, arthroscopic rotator‐cuff repair; AUC of ROC, area under curve of receiver operating characteristic; CNN, convolutional neural network; DL, deep learning; GB, gradient boosting; MRI, magnetic resonance imaging; PAN, Path Aggregation Network; RCT, rotator cuff tear; RF, random forest; SD, standard deviation; SITC, supraspinatus/infraspinatus tendon complex; SLAP, superior labrum anterior to posterior; SVM, support vector machine; TSS, tears of the subscapularis; XGBoost, Extreme Gradient Boosting; YOLOv8, you only look once.

Studies were screened based on the type of musculoskeletal disorder under investigation. This screening of conditions included supraspinatus/infraspinatus tendon complex (SITC) injuries, RCTs, traumatic shoulder injury, osteoarthritis, fracture, post‐traumatic dislocation, avascular necrosis, rheumatoid arthritis, osteonecrosis, anterosuperior impingement, SLAP lesions and traumatic arthropathy.

Detailed information regarding the specific interventions employed in the studies was collated. This comprised data on whether the intervention was rotator cuff repair (RCR), a/r TSA (anatomical/reverse), anterior shoulder instability (ASI) surgery, or other surgical or non‐surgical treatments.

Specific ML models utilized in the studies, including random forest (RF), support vector machines (SVMs), neural networks and others (Table 1, Supporting Information: Tables S1 and S2), were documented.

Outcome metrics were collected to evaluate model performance. Discriminative capacity between conditions was assessed using the AUROC or AUC. Accuracy was used as a key indicator of predictive performance. Precision, recall and specificity were analyzed, with the F1 Score providing a balanced measure of these metrics. The Brier Score and C‐Statistic were included to evaluate probabilistic prediction accuracy and overall model performance. Outcome metrics were considered for analysis only if reported by at least three of the 33 selected studies.

### Study risk of bias assessment

Two independent reviewers assessed the methodological quality of included studies, a third reviewer was consulted if discrepancies were not resolved by discussion. Two different tools, the JBI Critical Appraisal tool [42], and the ROBINS‐I [16] tool were chosen for the methodological quality assessment of the included studies, for case‐series and case–control studies, respectively. The tools were used to assess the risk of bias as high, moderate, or low.

## RESULTS

### Study selection

Figure 1 displays the PRISMA flow chart, which depicts the steps of selection of the included articles. 1329 records were retrieved and only 149 articles were selected for full‐text screening. Finally, a total of 33 articles were included in the systematic review.

### Study characteristics

All included studies involved the application of ML models to real patient information sourced from databases collected either retrospectively or prospectively with moderate to large sample sizes (100‐74697). In total, information on 179,778 patients was included in this systematic review.

#### Diagnostic models

Of the 33 included studies, 9 were identified as ‘Diagnostic’ [2, 15, 17, 23, 24, 27, 36, 37, 39], due to the utilisation of AI for interpreting diagnostic imagery and questionnaires, thereby improving shoulder injury diagnostics (Table 1). RCTs emerged as the predominant musculoskeletal disorder under investigation, employing magnetic resonance imaging (MRI) scans [23, 24], questionnaires and examination findings [27] for ML model training. Studies [17, 39] focused on diagnosing subscapularis tendon tears, utilizing radiological images and clinical information from physical exam to train the ML data sets [39]. SITC, Supraspinatus tear and SLAP lesion were respectively studied [2, 15, 37]; MRI [15, 37] and X‐Ray [2] images were used to train the ML models.

#### Predictive models

Among the studies, 18 were designated as ‘Predictive’ [1, 4, 5, 7, 8, 9, 10, 14, 18, 19, 20, 29, 31, 32, 35, 38, 43, 49] for their application of ML models in forecasting clinical outcomes (Supporting Information: Table S1). TSA was identified as the primary surgical procedure, with its outcomes explored in 13 studies [4, 5, 7, 9, 10, 14, 18, 19, 20, 31, 32, 35, 38]. These studies trained AI using clinical data, including preoperative information, medical comorbidities and demographic factors, to predict postoperative results, including complications and internal rotation (IR) [20]. Studies [1, 8, 29, 43] described AI's predictive capacity following anterior RCR. One study [1] focused on AI‐prediction of the non‐achievement of the minimal clinically important difference of disability post‐RC repair. Another study [8] utilized intraoperative images to train AI in predicting retear probabilities of RCR, whereas Longo et al. [29] focused on PROMs and clinical data for postoperative outcome ML forecasts. Shinohara et al. [43] concentrated on predicting postoperative complications using AI with clinical data sets, while Vassalou et al. [49] used two ML models to predict the long‐term complete pain resolution in patients undergoing ultrasound‐guided percutaneous irrigation of calcific tendinopathy.

#### Surgical models

Within this review, six studies were categorised as ‘Surgical’ [6, 21, 45, 46, 47, 48], leveraging AI to analyse radiological images from prior surgeries to improve the accuracy of revision surgery and to ascertain the advisability of intervention, thus facilitating improved surgeon‐patient communication (Supporting Information: Table S2). Researchers [6, 21, 46, 47, 48] developed AI capable of examining post‐operative radiological images (X‐Ray) to identify the manufacturer and model of TSA implants, speeding up the treatment process and avoiding unexpected intraoperative challenges and excessive health care costs. Simmons et al. [45] developed and trained an ML‐based clinical decision support tool (CDST) with the aim of enhancing evidence‐based decision‐making and improving preoperative patient counselling, specifically for TSA.

### Risk of bias in studies

**Figure 2:**
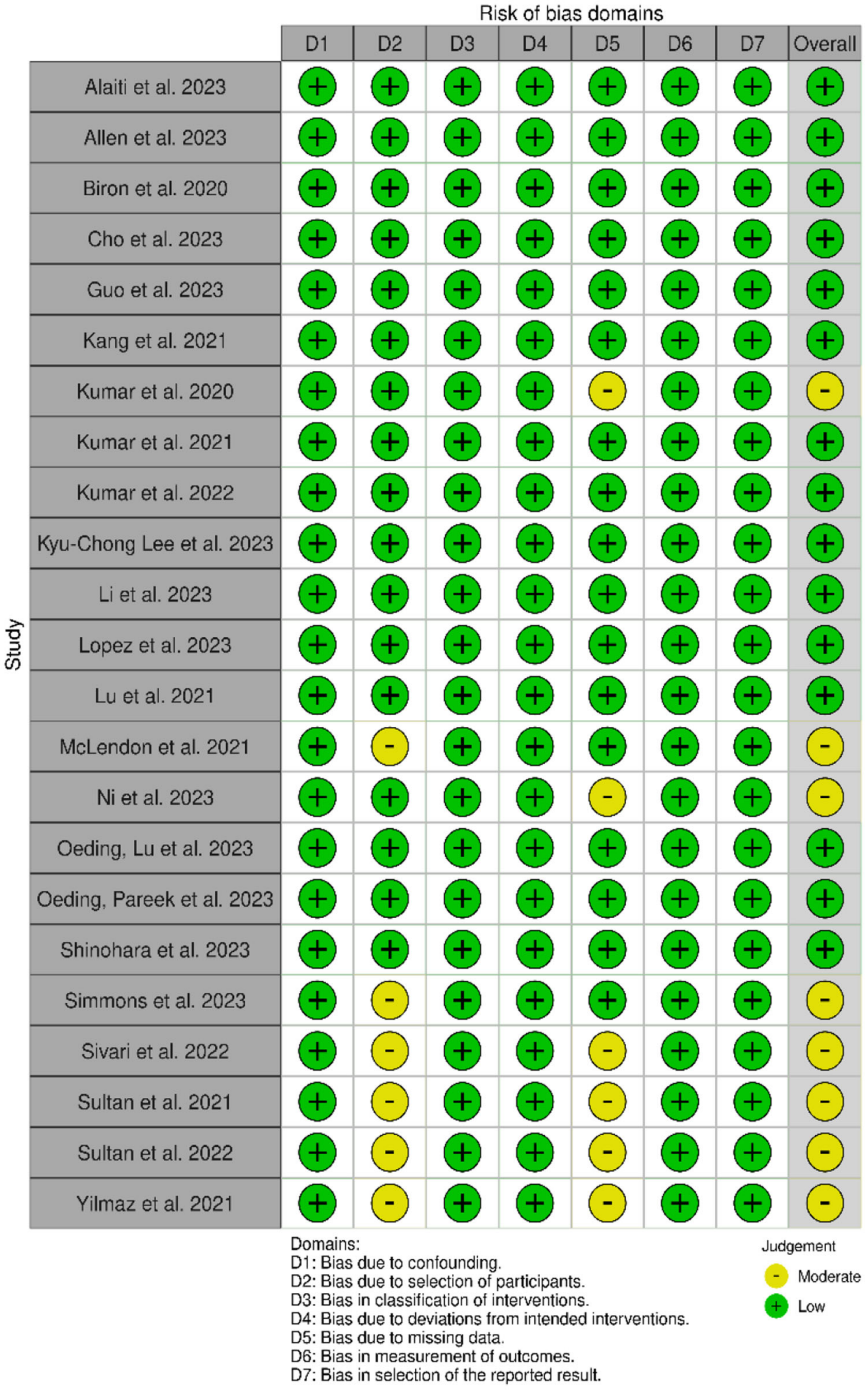
ROBINS‐I risk of bias evaluation of included case–control studies.

**Figure 3:**
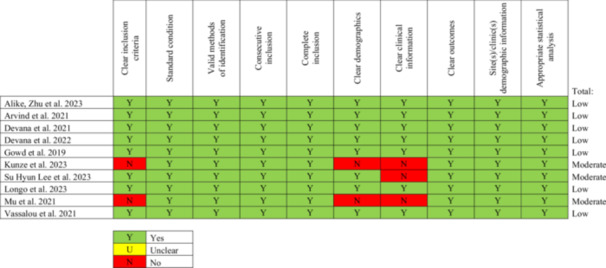
Joanna Briggs Institute critical appraisal risk of bias of included case‐series studies.

Two different tools were used to assess the risk of bias: the ROBINS‐I tool for case‐control studies (Figure 2) [1, 4, 6, 7, 8, 15, 17, 18, 19, 20, 23, 27, 31, 32, 35, 37, 38, 39, 43, 45, 46, 47, 48] and the JBI Critical Appraisal tool for case‐series studies (Figure 3) [2, 5, 9, 10, 14, 21, 24, 29, 36, 49]. They are reported in Figures 2 and 3, respectively.

Almost all the studies obtained a low risk of bias concerning clear outcomes, highlighting the rigorous mathematical foundation inherent within the field of AI. This framework quantitatively assesses the output variable with marked scientific precision. The inclusion criteria of participants and their clinical information were subject to a high risk of bias, due to the nature of the studies that did not focus on a follow‐up of the patients, but more on the prediction capability of the ML models.

### Results of individual studies

Detailed narrative descriptions of the results of the included studies can be found in Appendix B. Results have been divided according to the table they belong to.

#### Diagnostic

Seven of the included studies [2, 15, 17, 23, 24, 27, 39] utilized AI algorithms for the prediction of rotator cuff lesions, some evaluated single tendon lesions such as supraspinatus and subscapularis tears, while others considered a diagnosis of SITC injuries or RCTs in general (Table 1). AUC results ranged from 0.812 to 0.94, and one study [24] reported a sensitivity of 97% for the detection of RCTs.

One study [37] evaluated the identification of SLAP lesions and reported AUC values ranging from 0.92 to 0.98.

Finally, another study [36] evaluated the capacity of automatic shoulder joint segmentation from MRI images and reported a Dice coefficient of 0.91 ± 0.02.

#### Predictive

Nine studies [4, 5, 7, 9, 14, 19, 31, 35, 38] evaluated the prediction of a variety of parameters related to patients undergoing shoulder arthroplasty (Supporting Information: Table S1). These parameters include fairness and accuracy of CDSTs, unplanned readmissions, patient selection for outpatient surgery, complications and early dislocation. An RF model yielded an AUC of 0.77 for the prediction of patients suitable for short stays [31]. One study [31] reported an AUC of 0.851 for accurate prediction of non‐home discharge, another [19] showed a range of AUROC values from 0.79 to 0.97 in the prediction of clinical outcomes following reverse total shoulder arthroplasty (rTSA). While a study by Oeding et al. [38] focused on predicting early dislocation after rTSA and found that an extreme gradient boosting algorithm achieved a recall of 0.84.

Four studies [1, 8, 29, 43] predicted outcomes following RCR. An AUC value ranging from 0.58 to 0.68 was found for the prediction of PROMs [1], and an AUC range of 0.87–0.92 was found for the prediction of retear rate [8, 43]. One study [29] instead reported an accuracy of 46.5% for AI prediction of postoperative outcomes following RCT.

One study [49] examined the outcomes of ultrasound‐guided percutaneous irrigation of calcific tendinopathy, and the XGBoost model achieved an AUC of 69.2% for the model including VAS data at 1 week.

#### Surgical

Five studies [6, 21, 46, 47, 48] evaluated the identification of shoulder implant model following TSA from radiographs (Supporting Information: Table S2). Accuracies ranged from 89.90% to 97.20%, and one study reported sensitivities ranging from 0.80 to 1.00. Generally, tudies [6, 47, 48] report increased accuracy of implant identification with the application of data augmentation.

One study [45] evaluated a predictive CDST for anatomic and reverse TSA using ML and demonstrated generally accurate predictions with AUROC values between 0.70 and 0.97.

## DISCUSSION

The use of AI algorithms for the diagnosis and treatment of shoulder pathology is quickly evolving. Studies report high AUC values for diagnosing RCTs, SLAP lesions, and joint segmentation, along with strong accuracy in predicting PROMs, complications and treatment outcomes. Implant recognition from imaging also showed high sensitivity.

Findings reveal that AI significantly enhances the accuracy of diagnosis, via identification of RCTs from MRI imaging and automatic segmentation of the shoulder joint [12, 25, 44]. A study aimed at improving the bone structure and shoulder joint segmentation, provided an AI algorithm with a data set of MRI images and successfully demonstrated improvement in capabilities, that may eventually be applied to more precise injury diagnosis [36]. AI also demonstrates significant efficacy in forecasting clinical outcomes, readmissions, outpatient suitability, retear rates and post‐surgery complications [6, 21, 45, 46, 47, 48]. However, the performance varies across specific patient cohorts and outcomes, but more importantly, a handful of studies show large variability in accuracy across algorithms despite working on the same data sets [2, 3, 23]. Considering these findings, AI algorithms may represent a tool to facilitate and anticipate diagnosis, leading to more precise and better treatment planning that may also improve outcomes [34]. However, more research regarding a possibly standardized selection of algorithms with higher accuracy of diagnosis over less efficient counterparts is still not available. Additionally, their application in the prediction of surgical or patient outcomes can also better prepare clinicians to deal with specific adverse events or other patient complications [9, 14].

AI has improved surgical planning for shoulder disorders, accurately identifying implants, classifying manufacturers, enhancing precision with data augmentation and predicting TSA outcomes [6, 47, 48]. These findings can be clinically applied to improve preoperative planning by allowing surgeons to create more precise and individualized surgical plans based on detailed AI analysis of patient‐specific imaging and data. Optimizing implant selection may also be achieved by utilizing AI to match the best‐suited implants to a patient's unique anatomy and condition, potentially increasing the longevity and effectiveness of the implants [22]. One study concluded that ML algorithms had good to excellent accuracy for predicting within one size of the final components used for TKA [22], currently however, studies explore implant recognition rather than selection in case of shoulder arthroplasty [6, 22, 46, 48].

Of note is the emergence of the RF algorithm as a prevalent ML model, which was featured in 13 of the included studies [5, 7, 13, 27, 29, 31]. Its prevalence echoes its possible utility in medical data analysis. This algorithm is often able to produce accurate diagnoses and to predict treatment outcomes for shoulder disorders by analysing complex data sets, including imaging and clinical variables [5, 7, 27, 29, 31]. Nevertheless, there is a discrepancy within included studies; in one case the RF model accurately identified patients suitable for short stays [7], while in another evaluating prediction of outcomes of RCT patients RF had suboptimal performance [28]. Thus, in order to achieve more consistent results, it is crucial to improve the rigour of the studies analysing the RF algorithm and those working on improvements that may lead to improved application of this algorithm in the near future.

AI's application in improving diagnostic precision, particularly in interpreting MRIs and X‐rays, mirrors wider healthcare trends where AI acts as a decision‐support tool. This is exemplified by the CML‐DL model's superior performance in diagnosing SITC injuries [2] and the 2D Convolutional Neural Network model's high diagnostic performance for supraspinatus tears [15]. In predictive analytics, the review's findings suggest that ML models, especially RF classifier and XGBoost algorithm, are more effective in forecasting clinical outcomes post‐surgery compared to other algorithms [39]. It is relevant to mention that amongst consecutive studies and independent studies, there is still variation in AUC values obtained using the XGBoost algorithm [9, 10, 18, 19]. This suggests that fine‐tuning is required regardless of AI's increasing role in personalized medicine.

It is relevant to mention that in the included studies, the utilisation of the AUROC in 23 studies as a performance measure aligns with its use in medical literature evaluating AI algorithms. It is a relevant figure in evaluating the diagnostic and predictive accuracy of AI and ML models. It is also a reliable parameter to compare algorithms, and its wide use in the studies underlines the importance of improving the standardized measures to evaluate novel algorithms.

Despite the ongoing necessity of fine‐tuning of these algorithms they are already being applied in the field of orthopaedics. One study [11], created ML‐based prognostic model for patients undergoing hip arthroscopy. In this study, prognostic models were used to predict survivorship and the need for repeat surgeries which were both adapted into web‐based tools to assist the physician with shared decision making [11]. As specified in the article, this methodology may be used to create other predictive analytics models in different realms of orthopaedic surgery, contributing to the evolution from evidence‐based medicine to personalized medicine.

More standardized, high‐level research is needed to assess the accuracy and reliability of AI algorithms for specific clinical applications. This requires a deeper understanding of AI and ML in medical research and may involve shifting from patient‐centred to AI‐centred research, a departure from conventional medical studies [41]. While the former focuses on assessing the effectiveness of treatments, the latter aims to ascertain the accuracy of a machine, thus necessitating alternative methods for classifying studies [26, 41]. Despite this gap in literature and considering the present results, the integration of AI tools in orthopaedic practice is a possibility for the present and for the future; however, their application requires ongoing fine‐tuning and improvement from all points of view, from efficacy to approved standardisations and classifications of algorithms. From a policy perspective, it is still necessary to establish standards and guidelines for AI applications in healthcare, ensuring patient safety, data privacy and ethical use of AI. Future research should focus on prospective, multi‐centre trials to validate the effectiveness of AI tools in diverse clinical settings.

Overall, the present study ensured good methodological rigour by systematically screening studies based on the type of shoulder disorder, allowing for meaningful comparisons of AI models' efficacy across various pathologies. Detailed intervention data were collected, enabling an assessment of how different treatments influenced outcomes. Furthermore, the specific models used were documented to facilitate a comparative analysis of their effectiveness. Finally, to enhance the robustness and relevance of findings, outcome metrics were included only if reported by at least three of the 33 selected studies, ensuring a reliable basis for evaluation.

Despite such rigour, this review presents a variety of limitations. The diverse methodologies, outcome measures, varying levels of evidence, and different ML models affect the generalizability of the present findings. Additionally, these factors have made it impossible to perform meta‐analysis of the presented results. Despite their differences, the included studies and the presentation of their results are relevant thanks to the exploration of various aspects of the clinical application of AI in the management of shoulder disorders. A key limitation is the reliance on retrospective data, which may introduce bias. However, since AI training remains unaffected by data origin, the results still provide valuable insights into AI applications and accuracy.

Overall, the present systematic review illustrates AI's growing influence in the management of shoulder musculoskeletal disorders, and details its possible applications to diagnosis, prediction and surgical treatment. Although beneficial, integrating these technologies into everyday clinical practice demands careful evaluation of their limitations, ethical considerations, and the necessity for new and updated guidelines.

## CONCLUSION

AI application enables forecasting of clinical outcomes, permits refined diagnostic evaluation, and increases surgical accuracy. While promising, the translation of these technologies into routine clinical practice requires careful consideration.

## AUTHOR CONTRIBUTIONS

**Umile Giuseppe Longo**: Conceptualization; methodology; software validation; formal analysis; investigation; data curation; writing—review and editing; supervision; project administration. **Kristian Samuelsson**: Conceptualization; methodology; software validation; formal analysis; investigation; data curation; writing—review and editing; supervision. **Martina Marino**: Methodology; software validation; formal analysis; investigation; data curation; writing—original draft preparation; writing—review and editing; visualization. **Guido Nicodemi**: Methodology; software validation; formal analysis; investigation; data curation; writing—original draft preparation; writing—review and editing; visualization. **Matteo Giuseppe Pisani**: Methodology; software validation; formal analysis; investigation; data curation; writing—original draft preparation; writing—review and editing; visualization. **Jacob F. Oeding**: Methodology; software validation; formal analysis; investigation; data curation; writing—review and editing; supervision. **Cristophe Ley**: Methodology; software validation; formal analysis; investigation; data curation; writing—review and editing; supervision. **Rocco Papalia**: Methodology; software validation; formal analysis; investigation; data curation; writing—review and editing.

## CONFLICT OF INTEREST STATEMENT

Kristian Samuelsson is a Member of the Board of Directors of Getinge AB (publ) and medtech advisor to Carl Bennet AB. Other authors declare no conflicts of interest.

## ETHICS STATEMENT

The ethics statement is not available.

## Supporting information

Table S1: Results of Studies Evaluating Predictive Artificial Intelligence Algorithms.

Table S2: Results of Studies Evaluating Artificial Intelligence Algorithms for Surgical Applications.

## Data Availability

All data generated or analysed during this study are included in this published article and its Supporting Information files.

## APPENDIX A SEARCH STRATEGIES FOR EACH DATABASE

The search strategies were designed using a combination of Medical Subject Headings (MeSH) terms and free‐text terms. The specific syntax for each database is presented below.

**PubMed/MEDLINE Search Strategy**

((“Shoulder Resurfacing”[MeSH]) OR (“Shoulder Arthroscopy”[MeSH]) OR (“Shoulder Replacement”[MeSH]) OR (“Shoulder Arthroplasty”[MeSH]) OR (“Rotator Cuff Repair”[MeSH]) OR (“Reverse Shoulder Replacement”[MeSH]) OR (“Shoulder Surgery”[MeSH]) OR (“Shoulder Operation”[MeSH]))

AND

((“Artificial Intelligence”[MeSH]) OR (“Surgical Artificial Intelligence”[Title/Abstract]) OR (“Machine Learning”[MeSH]) OR (“Algorithm”[Title/Abstract]) OR (“AI”[Title/Abstract]))

AND

((“Complications”[MeSH]) OR (“Length of Stay”[MeSH]) OR (“Hospitalization”[MeSH]) OR (“Costs”[MeSH]) OR (“Economic Analyses”[MeSH]) OR (“Functional Outcomes”[MeSH]) OR (“Range of Motion”[MeSH]) OR (“Revision”[MeSH]) OR (“Revision Rate”[Title/Abstract]) OR (“Surgical Technique”[Title/Abstract]) OR ((“Blood”[MeSH]) AND (“Transfusion”[MeSH]) OR (“Loss”[Title/Abstract]))).

**SCOPUS Search Strategy**

(TITLE‐ABS((“Shoulder Resurfacing”) OR (“Shoulder Arthroscopy”) OR (“Shoulder Replacement”) OR (“Shoulder Arthroplasty”) OR (“Rotator Cuff Repair”) OR (“Reverse Shoulder Replacement”) OR (“Shoulder Surgery”) OR (“Shoulder Operation”)))

AND

(TITLE‐ABS((“Artificial Intelligence”) OR (“Surgical Artificial Intelligence”) OR (“Machine Learning”) OR (“Algorithm”) OR (“AI”)))

AND

(TITLE‐ABS((“Complications”) OR (“Length of Stay”) OR (“Hospitalization”) OR (“Costs”) OR (“Economic Analyses”) OR (“Functional Outcomes”) OR (“Range of Motion”) OR (“Revision”) OR (“Revision Rate”) OR (“Surgical Technique”) OR ((“Blood“ AND “Transfusion”) OR (“Loss”)))).

**Cochrane Database of Systematic Reviews Search Strategy**

((“Shoulder Resurfacing”) OR (“Shoulder Arthroscopy”) OR (“Shoulder Replacement”) OR (“Shoulder Arthroplasty”) OR (“Rotator Cuff Repair”) OR (“Reverse Shoulder Replacement”) OR (“Shoulder Surgery”) OR (“Shoulder Operation”))

AND

((“Artificial Intelligence”) OR (“Surgical Artificial Intelligence”) OR (“Machine Learning”) OR (“Algorithm”) OR (“AI”))

AND

((“Complications”) OR (“Length of Stay”) OR (“Hospitalization”) OR (“Costs”) OR (“Economic Analyses”) OR (“Functional Outcomes”) OR (“Range of Motion”) OR (“Revision”) OR (“Revision Rate”) OR (“Surgical Technique”) OR ((“Blood” AND “Transfusion”) OR (“Loss”))).

**EMBASE Search Strategy**

(‘Shoulder Resurfacing’/exp OR ‘Shoulder Arthroscopy’/exp OR ‘Shoulder Replacement’/exp OR ‘Shoulder Arthroplasty’/exp OR ‘Rotator Cuff Repair’/exp OR ‘Reverse Shoulder Replacement’/exp OR ‘Shoulder Surgery’/exp OR ‘Shoulder Operation’/exp)

AND

(‘Artificial Intelligence’/exp OR ‘Surgical Artificial Intelligence’:ti,ab OR ‘Machine Learning’/exp OR ‘Algorithm’:ti,ab OR ‘AI’:ti,ab)

AND

(‘Complications’/exp OR ‘Length of Stay’/exp OR ‘Hospitalization’/exp OR ‘Costs’/exp OR ‘Economic Analyses’/exp OR ‘Functional Outcomes’/exp OR ‘Range of Motion’/exp OR ‘Revision’/exp OR ‘Revision Rate’:ti,ab OR ‘Surgical Technique’:ti,ab OR ((‘Blood’/exp AND ‘Transfusion’/exp) OR ‘Loss’:ti,ab)).

## APPENDIX B NARRATIVE DESCRIPTIONS OF TABLE 1 AND SUPPORTING INFORMATION: TABLES S1 AND S2 RESULTS

*Diagnostic*

Alike et al. [3] developed an ensemble clinical machine learning‐deep learning (CML‐DL) model, integrating deep visual features with clinical data to predict SITC injuries (Table 1). Evaluated across 974 patients, the model demonstrated superior performance with sensitivity, specificity, accuracy and AUC of 0.880, 0.812, 0.836 and 0.902, respectively, outperforming individual models and significantly enhancing junior physicians' diagnostic capabilities.

Guo et al. [15] introduced a 2D convolutional neural network (CNN) model for automated diagnosis of supraspinatus tears (STs) using MRI images. The model's high diagnostic performance, with AUCs of 0.921 and 0.882 for surgery and internal test sets, respectively, suggests its potential to assist less experienced radiologists, particularly in community settings lacking expert consultation.

Kang et al.'s [17] deep learning (DL) algorithm focused on evaluating subscapularis tendon tears using axillary lateral shoulder radiographs, achieving an AUC of 0.83 for arthroscopic findings and 0.82 for MRI findings. The algorithm's moderate accuracy and high sensitivity suggest its utility in the initial assessment of subscapularis tendon integrity.

Lee et al. [23] developed a DL model for automatic detection of RCT across all MRI planes, achieving an AUC of 0.94 and indicating high effectiveness in detecting RCT, outperforming single‐plane analyses.

Lee et al. [24] employed a 3D U‐Net CNN to detect, segment, and visualize RCT lesions in 3D MRI scans, demonstrating high accuracy and efficiency with a Dice coefficient score of 94.3% and sensitivity of 97.1%.

Li et. al.'s [27] study on predicting RCT in outpatients using various ML algorithms highlighted the XGBoost model's superior performance, with an accuracy of 0.85 and AUC of 0.92, identifying critical clinical features for early diagnosis.

Mu et. al. [36] focused on the automatic segmentation of shoulder joint MRI images using CNNs, showing accurate segmentation capability with a Dice Coefficient of 0.91 ± 0.02.

Ni et. al. [37] evaluated the SLAP‐Net model for identifying SLAP lesions, demonstrating high diagnostic performance comparable to senior radiologists, with AUCs of 0.98 and 0.92 across different data sets.

Oeding et al. [38] developed a ML model to predict subscapularis tears using pre‐operative imaging data, achieving excellent predictive performance with a C‐statistic of 0.84 and accuracy of 0.85, indicating its potential in clinical decision‐making.

*Predictive*

Alaiti et al. [1] investigated the use of ML to predict patient‐reported outcomes following rotator cuff surgery (Supporting Information: Table S1). Analysing data from 474 patients, they found AUC values ranging from 0.58 to 0.68, indicating moderate predictive accuracy.

Allen et al. [4] assessed the fairness and accuracy of ML‐based CDSTs post‐shoulder arthroplasty across demographic attributes. They analysed data from 8,280 patients and reported minor differences in prediction errors across different demographics.

Arvind et al. [5] focused on predicting unplanned readmission following TSA. The study highlighted the RF classifier's superior predictive capability with a c‐statistic of 0.74.

Biron et al. [7] developed a ML model to assist in patient selection for outpatient TSA. Their RF model accurately identified patients suitable for short stays with an AUC of 0.77. Cho et al. [8] utilized DL algorithms to predict retear following arthroscopic rotator cuff repair. The DenseNet model showed a high level of accuracy with an AUC of 0.92.

Devana et al. [9] evaluated ML models for predicting complications and readmission after reverse TSA, highlighting the XGBoost model's high performance.

Gowd et al. [14] demonstrated the effectiveness of ML over traditional indices in predicting postoperative complications. Logistic regression showed the highest AUC values for various complications.

Kumar et al. [19] presented results on the accuracy of ML techniques in predicting clinical outcomes after shoulder arthroplasty. The study showed a range of AUROC values from 0.79 to 0.97.

Longo et al. [29] aimed to predict outcomes for RCT patients using ML, finding suboptimal performance with accuracies of 46.5% for logistic regression and 51.25% for RF.

Lopez et al. [31] evaluated ML models for predicting nonhome discharge after shoulder arthroplasty. The study reported an AUC of 0.851 for the ANN model and 0.788 for the boosted decision tree model.

McLendon et al. [35] investigated ML to predict improvement in shoulder arthroplasty, showing that Model 1 was the most sensitive in predicting post‐operative outcomes based on ASES score improvements.

Oeding et al. [38] focused on predicting early dislocation after reverse TSA. The extreme gradient boosting algorithm achieved a recall of 0.84.

Shinohara et al. [43] explored predicting re‐tear after arthroscopic RCT surgery. The LightGBM model showed superior performance with an AUC of 0.87.

Vassalou et al. [49] examined the outcomes of ultrasound‐guided percutaneous irrigation of calcific tendinopathy. The XGBoost model achieved an AUC of 69.2% for the model including VAS data at 1 week.

*Surgical*

Kunze et al. [21] developed an automated DL algorithm for the identification of shoulder arthroplasty implants from radiographs (Supporting Information: Table S2). The algorithm exhibited high accuracy (97.1%) in classifying implants from 8 manufacturers, encompassing 22 unique implants, with sensitivities ranging from 0.80 to 1.00. In single‐institution implant predictions, the model identified six specific implants with accuracies of 99.4% and sensitivity >0.97. This study underscores the potential utility of AI in preoperative planning for failed TSA cases.

Simmons et al. [45] evaluated a predictive CDST for anatomic and reverse TSA using ML. This tool underwent external validation on 243 patients, demonstrating generally accurate predictions with AUROC values between 0.70 and 0.97. This study emphasizes the importance of understanding the limitations of ML‐based CDSTs in orthopaedic surgery, particularly in decision‐making and patient counselling.

In Sivari et al. [46], the focus was on classifying shoulder implant manufacturers using hybrid ML models. The study reported that the DenseNet201 + Logistic Regression model achieved the highest accuracy (95.07%), indicating the effectiveness of hybrid AI approaches in this domain.

Sultan et al. [48] compared the accuracies of various models for shoulder implant classification, both with and without data augmentation. The study presented a comprehensive comparison across different CNNs models, showing varied performance metrics under different conditions like data augmentation and Random Image Augmentation.

Another study by Sultan et al. [47] reported on the IMFC‐Net model, a novel AI approach for shoulder implant classification. The model achieved an accuracy of 89.09% without data augmentation and showed improved performance with Rotational Invariant Augmentation, suggesting the importance of data augmentation techniques in enhancing AI model accuracy.

Yılmaz et al. [50] evaluated the performance of various DL models for shoulder implant manufacturer and model detection. The proposed model in this study demonstrated high accuracy (97.20% without data augmentation and 96.31% with it), outperforming other models like ResNet‐50 and Dark‐Net‐53.
